# An unusual cause of lagophthalmos

**DOI:** 10.4103/0974-620X.60020

**Published:** 2010

**Authors:** G.I. Nambi, B. Beck, A.K. Gupta

**Affiliations:** Department of Plastic and Reconstructive Surgery, Christian Medical College, Vellore, Tamilnadu, India

**Figure 1:**
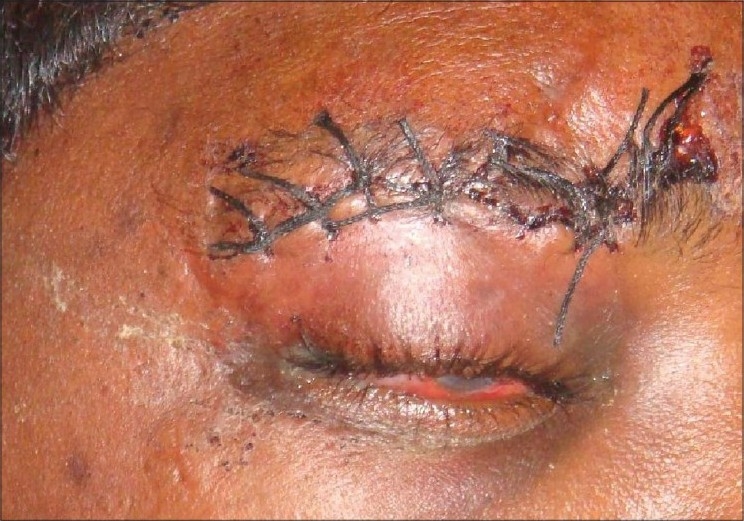
Irregular hemostatic sutures of the eye brow causing lagophthalmus

**Figure 2:**
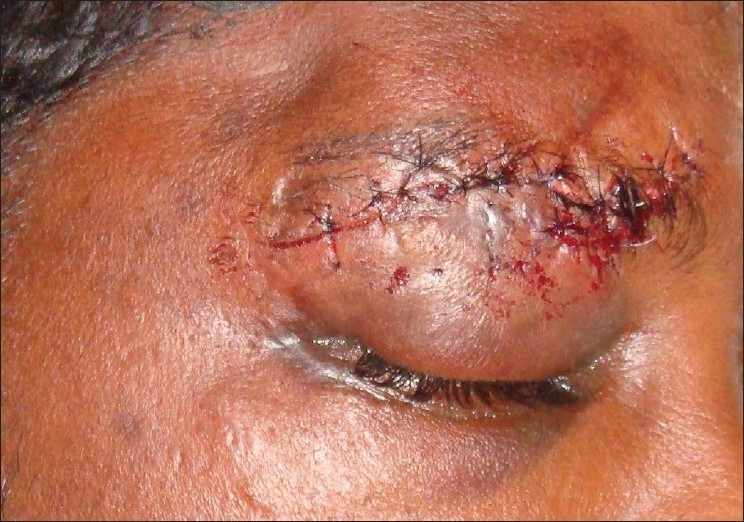
Correction of lagophthalmus after revision of hemostatic suture

A 25-year-old youth presented with inability to close his right upper eyelid completely since two days following suturing of an eyebrow laceration, which was done elsewhere. The wound was due to impact against the handle bar following a bicycling accident. On examination, there was wound in his right eye brow, which was closed with continuous interlocking sutures [Figure 1], the bites of which were away from the wound margins. Further, the suture bites of the lower wound margins were incorporating the skin and subcutaneous tissue of the upper eyelid resulting in traction of the eyelid and causing lagophthalmos. There was periorbital edema and subconjunctival hemorrhage with normal visual acuity and the opposite eye was normal. A decision was made to revise the suturing so as to correct the ′iatrogenic lagophthalmos′. Under local anesthesia, with aseptic precautions, the primary suture was removed and the wound re-opened. As soon as the constricting sutures were removed and the eye lid released, it was seen fully closing the eyeball. After minimal debridement and saline irrigation, the wound was closed meticulously with proper alignment of the matching points and anatomical landmarks on either side of the wound with 4-0 vicryl sutures for the subcutaneous tissue and 5-0 nylon for the skin restoring the cosmetic appearance of the face and correcting the iatrogenically created lagophthalmos [Figure 2].

The word lagophthalmos is derived from the Greek word for hare eye.[1] It is a condition characterized by inability of the eyelid to close the eyeball completely. Lagophthalmos secondary to cranial nerve VII paresis or palsy may be idiopathic [Bell′s palsy], congenital [Moebius syndrome], or secondary to trauma [facial nerve injury], infection [Lyme′s disease], or iatrogenic [Resection of skull base tumors and blepharoplasty]. Lagophthalmos may also occur due to increased eye exposure [secondary to exophthalmos], shortened height of the upper eyelid, reduced tone of the orbital muscles, and palpaberal insufficiency.[2] Incomplete closure of the eyeball results in exposure of the cornea, dryness and a risk of keratitis, ulceration, perforation and blindness.[3,4]

Hemostatic sutures are applied in situations to control bleeding following acute trauma and are done by variety of suturing techniques such as continuous sutures with or with out inter locking and interrupted simple or mattress sutures. Though the techniques are not significant in hidden areas such as hair bearing scalp, they are significant in face which is a cosmetically sensitive area and in areas near landmarks such as side burns, eyebrow, lips, nasal ala or eyelids, where interrupted simple sutures are preferred with proper approximation of the matching points on either side of the wound so as to control the bleeding as well as to get a cosmetically acceptable scar. During the repair of eyelids after traumatic injury, thorough lavage of the wound with saline, avoiding excessive debridement and approximation of anatomical layers with fine suture materials help in preventing lid shortening where as during elective surgeries, avoiding the extension of the incision far too medially or laterally avoiding excessive removal of the eyelid muscle and orbital fat help in reducing the scar formation in postoperative period.

Excessive scarring of tissues causes contracture and lid shortening resulting in lagophthalmos. The condition is managed by:

- Conservative methods such as application of artificial tears (eye drops or ointment), physiotherapy, use of protective sunglasses and eye shields. The conservative methods are adopted when the condition is temporary or partial or till definitive surgical procedure is carried out.
- Surgery when there is complete inability to close the eyelids, with failed nonsurgical treatment to protect the cornea, and when there is possibility of long-term lid closure impairment.

Surgical methods to correct lagophthalmos including lateral tarsorraphy, temporalis fascial sling, ipsilateral and crossed facial nerve grafting, facial re-animation with gracilis neuro muscular free flap or application of gold weights[3] (for patients with facial palsy), can be carried out alone or in combination with reconstruction of other structures paralyzed in the face such as nose, cheek and lip. In our patient, although there was no anatomical loss of tissue or nerve injury, mere traction of the upper lid from thick suture bites of the eyebrow wound resulted in inability of the lid to close the eye and was corrected with meticulous re-suturing with proper alignment of the matching points on the wound edges and anatomical landmarks.
